# Altered Glucose Metabolism and Glucose Transporters in Systemic Organs After Bariatric Surgery

**DOI:** 10.3389/fendo.2022.937394

**Published:** 2022-07-14

**Authors:** Ju Hun Oh, Chan Woo Kang, Eun Kyung Wang, Jung Ho Nam, Soohyun Lee, Kyeong Hye Park, Eun Jig Lee, Arthur Cho, Cheol Ryong Ku

**Affiliations:** ^1^ Brain Korea 21 PLUS Project for Medical Science, Yonsei University, College of Medicine, Seoul, South Korea; ^2^ Department of Internal Medicine, Endocrinology, Institute of Endocrine Research, Yonsei University College of Medicine, Seoul, South Korea; ^3^ Department of Nuclear Medicine, Yonsei University College of Medicine, Seoul, South Korea; ^4^ Division of Endocrinology and Metabolism, Department of Internal Medicine, National Health Insurance Service Ilsan Hospital, Goyang, South Korea

**Keywords:** bariatric surgery, diabetes mellitus, glucose metabolism, glucose transporter, obesity

## Abstract

The Roux-en-Y gastric bypass (RYGB) is highly effective in the remission of obesity and associated diabetes. The mechanisms underlying obesity and type 2 diabetes mellitus remission after RYGB remain unclear. This study aimed to evaluate the changes in continuous dynamic FDG uptake patterns after RYGB and examine the correlation between glucose metabolism and its transporters in variable endocrine organs using ^18^F-fluoro-2-deoxyglucose positron emission tomography images. Increased glucose metabolism in specific organs, such as the small intestine and various fat tissues, is closely associated with improved glycemic control after RYGB. In Otsuka Long-Evans Tokushima Fatty rats fed with high-fat diets, RYGB operation increases intestine glucose transporter expression and various fat tissues’ glucose transporters, which are not affected by insulin. The fasting glucose decrement was significantly associated with RYGB, sustained weight loss, post-RYGB oral glucose tolerance test (OGTT) area under the curve (AUC), glucose transporter, or glycolytic enzymes in the small bowel and various fat tissues. High intestinal glucose metabolism and white adipose tissue-dependent glucose metabolism correlated with metabolic benefit after RYGB. These findings suggest that the newly developed glucose biodistribution accompanied by increased glucose transporters is a mechanism associated with the systemic effect of RYGB.

## Introduction

Bariatric surgery provides a long-term solution for patients with severe obesity. Bariatric surgeries are effective for both substantial weight loss and improved glucose metabolism, resulting in the remission of type 2 diabetes mellitus (DM) (1–7), especially in patients with a history of diabetes for less than 5 years (4, 5, 8), and the effects are superior to conventional non-surgical diabetes therapy (4, 5). Among bariatric surgeries, Roux-en Y gastric bypass (RYGB) has the most powerful therapeutic effect on the resolution of DM and obesity. In most patients undergoing RYGB, improved glycemic control occurs initially within days before sustained weight loss, implicating the possibility of a weight-independent mechanism of bariatric surgery (9). In addition, several studies indicated that bariatric surgeries enhance the systemic hormone levels or signaling peptides, including GLP-1, GLP-2, CCK, 5-HT, PYY, and IGFBP-2 (10–16), causing alterations in bile acids and related pathways (17–19). However, to date, most studies mainly focused on the intestine after bariatric surgery (20–24), which cannot fully explain clinical improvement (16, 17, 25). Meanwhile, few studies focus on systemic metabolism change.

^18^F-fluoro-2-deoxyglucose (FDG) positron emission tomography (PET)/computed tomography (CT) is used in the diagnosis of cancer malignancies by capturing the high FDG uptake of cancer cells. Also, the FDG uptake pattern shows the biodistribution of physiological glucose disposal. We previously reported that after bariatric surgery, intestinal glycolysis and glucose excretion through the intestine were substantially increased, which contributed to the rapid and lasting beneficial effect on glycemic control that is predominantly weight loss independent (21, 24). Moreover, in this study, we observed the change of systemic glucose metabolism not only in robust intestinal FDG uptake but also in various organs. Although systemic increment of glucose metabolism was observed, whether this newly developed glucose metabolism is correlated with metabolic improvement after RYGB remains unclear.

This study aimed to evaluate the changes in continuous dynamic FDG uptake patterns after RYGB and examine the correlation between glucose metabolism and its transporters in variable endocrine organs.

## Methods

### Animals

All animal experiments were carried out under an Institutional Animal Care and Use Committee-approved protocol and institutional guidelines for the proper and humane use of animals. Four-week-old male Otsuka Long-Evans Tokushima Fatty (OLETF) rats were provided by the Tokushima Research Institute (Otsuka Pharmaceutical). OLETF rats were fed a high-fat diet (D12492, Research Diets, USA) for 16 weeks and were assigned to two types of surgery (RYGB: n=10 and sham surgery: n=4). The rats were housed at 22 ± 1°C under a 12-12 h light-dark cycle, with *ad libitum* access to water and food. Positron emission tomography (PET) was performed after an overnight fast before the surgery and 2, 4, and 8 weeks post-surgery and was processed as described below.

### Roux-En Y Gastric Bypass

RYGB was performed under anesthesia as described in a previous study (21). The length of the entire small intestine was measured from the Treitz ligament. The jejunum was cut 18 cm downstream from the Treitz ligament. In a jejuno-gastric anastomosis, an end-to-side gastrojejunostomy was performed, and the other end of the cut jejunum was anastomosed to the small intestine 20 cm distal to the gastrojejunostomy resulting in a long common limb.

### Sham Operation

The Sham group was used as a control. The sham surgery was performed as described in a previous study (21). Briefly, sham surgery consisted of laparotomy, a 1-cm enterotomy on the jejunum, and re-anastomosis without transection of jejunum and stomach.

### Micro-Positron Emission Tomography and Radiotracer Counting

μPET imaging was performed according to a previously published paper protocol (21, 24). After an overnight fast, approximately 1 mCi of 18F-fluoro-2-deoxyglucose (FDG) was injected *via* tail vein injection. One hour after FDG injection, a 10 min μPET scan was conducted using Inveon PET (Siemens Medical Solutions, Knoxville, KY, USA). The maximum standardized uptake value (SUVmax), averaged uptake value (SUVmean), SUVmax normalized to rodents’ weight (maxSUV), and SUVmean normalized to rodents’ weight (meanSUV) were measured with the volume of interest (VOI) drawn on multiple tissues on PET images using the pMOD software (version 4.3, Switzerland). The SUVmax of the VOI was measured using the following formula: (decay-corrected activity [kBq] per tissue volume [mL])/(injected 18F-FDG activity [kBq] per body mass [g]). Immediately after μPET scan, the rats were euthanized in a CO_2_ chamber, and tissues were excised, weighted, and subjected to gamma counting (Wallac Wizard 3” 1480 Gamma Counter; PerkinElmer, Akron, OH, USA). FDG tissue biodistribution data were decay-corrected according to the time of FDG injection and normalized to both the tissue weight (g) and radioactivity level. In counting FDG activity in the various tissues, the counts per minute were normalized to the dose of radioactivity injected.

### Quantitative real-Time Polymerase Chain Reaction

Total RNA from tissues was isolated using the RNeasy Mini Kit (Qiagen, Germantown, MD, USA) according to the manufacturer’s protocol. cDNA was synthesized using the ReverTra Ace (Toyobo, Osaka, Japan). qRT-PCR was performed using the ABI StepOne Plus Real-Time PCR machine (Applied Biosystems, Foster City, CA, USA). The primers used in this study are listed in **Table S1**. The relative transcriptional expression of target genes was evaluated by Eq. 2^-ΔCt^ (ΔCt = Ct of target gene minus Ct of reference gene of each organ tissue).

### Statistical Analysis

All data were expressed as means ± SEM. Statistical analyses were performed using the Prism software version 4.0.0 (GraphPad, La Jolla, CA, USA). Each experiment was performed at least three times. All data were analyzed for statistical significance using the student’s t test. In addition, Spearman correlation analyses were performed for comparing post-op OGTT AUC, post-op fasting glucose, post-op weight, post-op FDG radioactivity, post-op glucose transporter and glucose metabolism enzyme mRNA level. Also, in the RYGB column, sham group was set as 0 and RYGB group was set as 1, and correlation analysis was performed to analyze the correlation between operation type and glucose biodistribution. Statistical significance was set at p < 0.05, and differences are indicated using asterisks (*p < 0.05, **p < 0.01, ***p < 0.001).

## Result

### FDG PET/CT Scanning Cases of Changes in Systemic Glucose Biodistribution After Gastrectomy in Human and RYGB in Rodents

**Figure 1:**
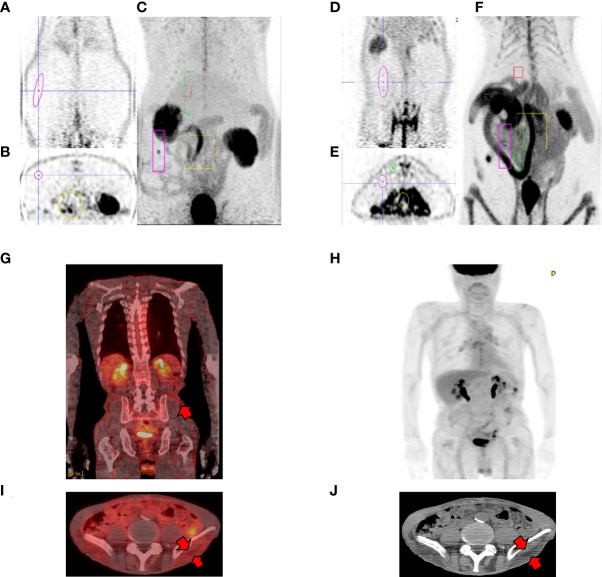
Representative PET cases of altered systemic glucose distribution. **(A-C)** pre-operative OLETFrat showing low FDG uptake in the subcutaneous fat layer. Note low FDG uptake in the bowel in MIP images. **(D-F)** post-operative OLETFrat showing increased FDG uptake in the subcutaneous layer 2 months after surgery. (**A**, **D** coronal PET, **B**, **E** axial PET, **C, F** maximum intensity projection (MIP) **(G-J)** Representative case of changes in biodistribution after gastrectomy. A 49-year-old male patient underwent Roux-en-Y gastroesophagectomy due to stomach cancer. FDG PET/CT was performed one year after surgery for surveillance. Fusion coronal **(G)** and axial fusion **(I)** images shows elevated FDG uptake in the corresponding subcutaneous fat layer (arrows, **J**).

Previous our study showed that increased intestinal glycolysis significantly correlated with reduction in HbA1c levels (21, 24). To further characterize the systemic glucose metabolism, we focused not only on small bowel but also the whole-body organs. We performed baseline and post-surgical FDG μPET in RYGB-operated rats. Pre-operated OLETF rats showed that low FDG uptake in the intestine and fat layer (**Figures 1A-C**). After RYGB surgery, it was confirmed that robust FDG uptake was observed in the intestine and subcutaneous layer (**Figures 1D-F**). We gained similar observations in patients who underwent FDG-PET/CT 1 year after Roux-en-Y gastroesophagectomy for gastric cancer. FDG-PET/CT showed elevated FDG uptake not only in small bowel but also in the subcutaneous fat layer (**Figures 1 G-J**), which is similar to observations from RYGB-operated rats (**Figures 1D-F**). Thus, these results indicated that newly developed glucose metabolism occurred in surgical groups after bariatric surgery.

### Altered Glucose Distribution After RYGB

When comparing PET images of patients who underwent bariatric surgery with PET images after RYGB with OLETF rats examined in this experiment, it was confirmed that, similar to humans, changes in systemic glucose metabolism and small intestine occurred in OLETF rats after RYGB (**Figure 1**).

**Figure 2:**
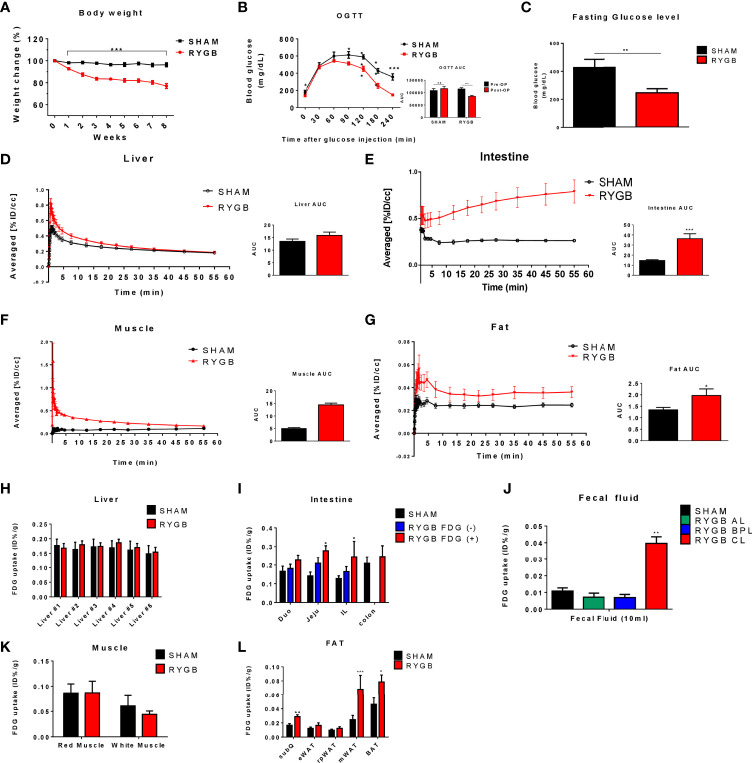
Altered glucose distribution after RYGB. Metabolic parameters in adult male Otsuka Ling-Evans Tokushima fatty (OLETF) rats showing weekly body weight change **(A)** measured every week over 8 weeks, oral glucose tolerance test (OGTT) data **(B)**, and fasting glucose level **(C)** in sham-operated and RYGB-operated rats. Representative 2-Deoxy-2-[18F]-fluoro-D-glucose (FDG)-PET image analysis in OLETF rats. OLETF rats Time Activity Curves (TAC) obtained from 60-min dynamic PET imaging and AUC graph of liver **(D)**, intestine **(E)**, muscle **(F)**, and mesenteric fat **(G)**. Liver #1~#6 denote Right Median Lobe (#1), Right Superior Lobe (#2), Right Median Lobe (#3), Left Median Lobe (#4), Left Lateral Lobe (#5), Caudate Lobe (#6), respectively **(H)**. FDG biodistribution analysis (gamma counting) in RYGB-operated and sham-operated rats in various organs, including the liver **(H)**, intestine **(I)**, intestinal lumen phosphate buffered saline (PBS) washing analysis **(J)**, muscle **(K)**, and various fat tissues **(L)**. Number of mice in all panels **(A–C)** (males; RYGB, 10; sham, 4). All data are presented as mean ± SEM, *p<0.05, **p<0.01, ***p<0.001 vs. sham.

To confirm our previous finding of the occurrence of a newly FDG uptake pattern and unexpected glucose retention and secretion in the intestine (21, 24), OLETF rats were fed a 60% high-fat diet for 16 weeks and were subjected to RYGB. All sham-operated rats gained or maintained weight, whereas those that were subjected to RYGB lost weight robustly and sustainably for 8 weeks (**Figure 2A**). We conducted OGTT and measured fasting glucose at 8 weeks post-surgery. As expected, compared to sham operation, RYGB resulted in improved glucose control (**Figures 2B, C**). Absolute body weight (702.1 ± 10.67 vs. 590.23 ± 6.25 g, p<0.01), fasting glucose levels (10.439 ± 0.212 vs. 7.849 ± 0.169 mmol/L, p<0.01), and body weight changes (%reduction compared with preoperative weight, +4.06% vs. – 25.2%, respectively), fasting glucose changes (%reduction compared with preoperative glucose, +15.9% vs. -42.6%, respectively) were significantly lower in RYGB-operated rats (**Figures 2A-C**).

To evaluate whether RYGB enhanced systemic glucose uptake, we performed baseline and post-RYGB dynamic FDG μPET. List-mode-time-of-flight data acquisition started immediately after the intravenous FDG administration and lasted for 1 h. Dynamic PET images were reconstructed, and six spherical regions of interest (ROI), each with 20 mm diameter, were placed on the six segments of each organ, avoiding any blood vessels. The averaged FDG activity in all six ROIs was extracted from the dynamic images to form a global Time Activity Curves (TAC) (**Figures 2D-G**). ^18^FDG distribution was characterized by rapid liver uptake, followed by clearance through the other organ, including the kidney and bladder (**Figures 1B**, **2D**). Rapid liver uptake of FDG was increased in RYGB rats but preceded a washout of FDG radioactivity over 60 min, and the difference between the two groups disappeared (**Figure 2D**). This phenomenon also occurred in muscles (**Figure 2F**). In contrast, compared to sham surgery rats, fat-specific radioactivity was still retained at 60 min in RYGB-operated rats (**Figure 2G**). Also, a previous study (21) showed that although FDG did not accumulate in the intestine of sham-surgery rats, the intestine of RYGB-treated rats showed a high accumulation of the FDG (**Figure 2E**).

To evaluate systemic changes in glucose metabolism after RYGB operation, multiple tissues were excised and subjected to gamma counting of FDG radioactivity immediately after μPET. FDG tissue biodistribution analysis showed that FDG uptake in the intestine and fat was higher in RYGB-operated rats compared with that in sham-operated rats, whereas no difference in the FDG uptake in the liver and muscle was observed (**Figures 2H, K**). Furthermore, excreted FDG into common limbs (CL) of the intestine lumen was increased in RYGB-operated CL compared to that of RYGB-operated alimentary limbs (AL), biliopancreatic limbs (BPL), and sham-operated rats (**Figure 2J**). Taken together, these data indicated that RYGB induced a newly glucose biodistribution through the intestine and fat tissue and that RYGB-induced improved glycemic control might be correlated with this newly glucose biodistribution.

### Expression of Glucose Transporters in Various Organs

**Figure 3:**
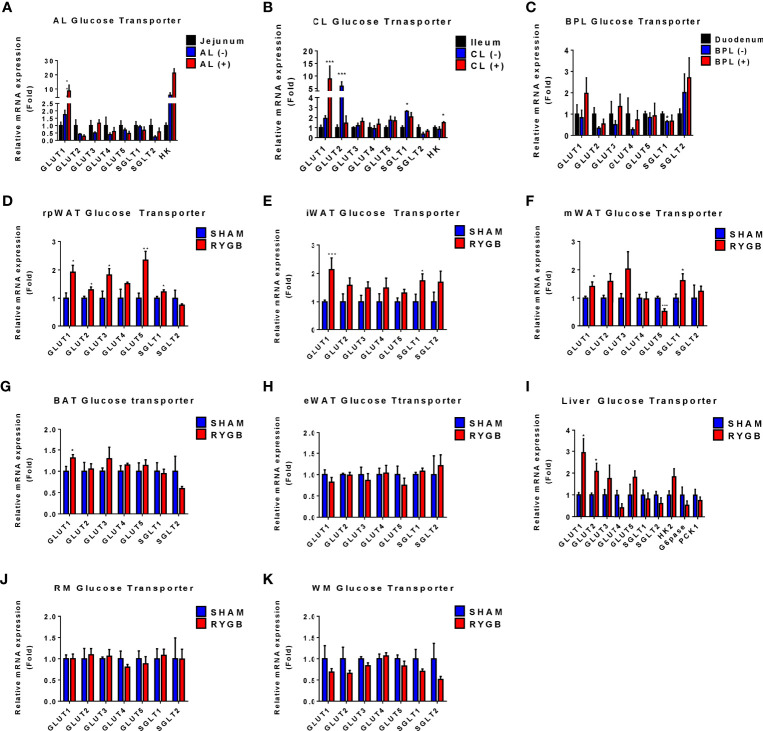
Expression of Glucose transporters in various organs. Subtypes of glucose transporters are altered in RYGB-operated OLETF rats. The expression of glucose transporter subtype in alimentary limbs (AL), common limb (CL), and biliopancreatic limb (BPL) of RYGB-operated rats and the corresponding limbs of sham-operated rats **(A-C)**. The fold change was calculated based on the corresponding sham-operated bowel (jejunum for the CL, duodenum for the biliopancreatic limbs (BPL), and ileum for the CL). (–) or (+) denotes FDG negativity or positivity of individual rats, respectively. The expression of glucose transporter subtype in retroperitoneal white adipose tissue (rpWAT) **(D)**, inguinal WAT (iWAT) **(E)**, mesentery WAT (mWAT) **(F)**, brown adipose tissue (BAT) **(G)**, epididymal WAT (eWAT) **(H)**, liver **(I)**, red muscle (RM) **(J)**, and white muscle (WM) **(K)**. The fold change was calculated based on the corresponding sham-operated rats’ organs. All data are presented as mean ± SEM, *p<0.05, **p<0.01, ***p<0.001 vs. sham.

Because systemic glucose uptake was observed in various tissues after RYGB, we evaluated which glucose uptake and glucose metabolism genes could influence this glucose homeostasis. Glucose transporter (GLUT) and enzyme genes were observed in tissues based on reverse transcription PCR (RT-PCR) analysis (**Figure 3**). In the intestine, we selected regions of each limb of the intestine with higher levels of FDG uptake (FDG +) or basal levels (FDG -) using gamma counting. Corresponding sections of sham-operated rats were used as controls. Consistent with previous study (21), in the AL of the intestine, GLUT1 and Hexokinase (HK) genes showed a sequential increase corresponding to FDG uptake increase patterns (sham jejunum, AL FDG (-), AL FDG (+)) (**Figure 3A**). Other GLUT genes were unchanged or decreased in sham and RYGB. Similarly, in CL, GLUT1 and HK genes were upregulated in FDG (+) AL (**Figure 3B**). However, no genes in the BPL of the intestine were differently regulated in RYGB rats (**Figure 3C**). We removed various fat tissues, including the inguinal white adipose tissue (iWAT), epididymal WAT (eWAT), brown adipose tissue (BAT), retroperitoneal WAT (rpWAT), and mesentery WAT (mWAT), to identify which fats contribute to the improvement of glucose homeostasis of RYGB-operated rats. In all fats, except for eWAT, GLUT1 expression was increased in RYGB-operated rats than that in sham rats (**Figures 3D-H**). In the rpWAT of RYGB-operated rats, GLUT1-5 expressions were significantly increased (**Figure 3D**). In the liver, GLUT1 and GLUT2 showed a significantly increased in RYGB-operation rats (**Figure 3I**). In accordance with above dynamic PET data, no genes were significantly altered in the muscle (**Figures 3J, K**). These results indicated that GLUT and HK are robustly associated with systemic and newly glucose biodistribution by RYGB.

### Correlation Analysis Between Glucose Distribution and Glucose Transporters

**Figure 4:**
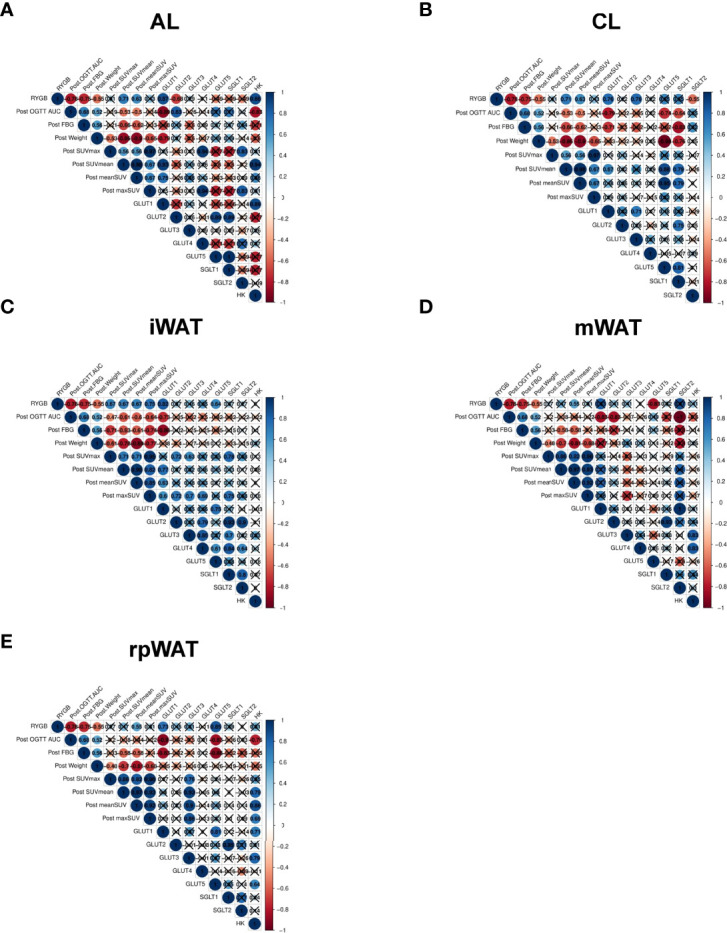
Correlation analysis between glucose distribution and glucose transporters Spearman’s correlation among post-operated OGTT AUC, post-operated weight, postoperated fasting glucose, operation type, the maximum standardized FDG uptake value (SUVmax), the averaged standardized FDG value, and each normalized to weight (maxSUV, meanSUV), and the expression levels of glucose transporters in **(A)** AL, **(B)** CL, **(C)** iWAT, **(D)** mWAT, and **(E)** rpWAT, representing significant FDG uptake in TAC images in RYGB-operated OLETF rats. Nonsignificant correlations (p-value > 0.05) are indicated by crosses.

To document the relationships between glucose uptake and metabolism related genes and system metabolic changes, we conducted correlation analysis. Operation type, post-op OGTT AUC, post-op fating glucose level, post-op weight, FDG radioactivity, and GLUT1-5 genes were used as multiple parameters for Pearson or Spearman correlation analysis. Since FDG uptake was increased in the AL of the intestine, CL of the intestine, and various adipose tissues among various organs, we conducted a correlation analysis with intestine and WAT (**Figure 4**). Correlation analysis showed that: (1) operation type, post-op AUC, post-op fasting glucose, and post-op weight were positively correlated (**Figures 4A-E**); (2) in the AL of intestine, RYGB surgery correlated with FDG radioactivity and GLUT1 expression, whereas post-op AUC, fasting glucose level, and weight were negatively associated with FDG uptake and GLUT1 expression (**Figure 4A**); (3) in the CL of intestine, RYGB surgery correlated with CL FDG radioactivity and CL GLUT1 expression, whereas post-op AUC, fasting glucose level, and weight were negatively associated with CL FDG uptake, CL GLUT1 expression, and SGLT1 expression (**Figure 4B**); (4) in iWAT, RYGB surgery correlated with iWAT FDG radioactivity, iWAT GLUT1 expression, and GLUT5 expression, whereas post-op AUC, fasting glucose level, and weight were negatively associated with iWAT FDG uptake and GLUT1 expression, while post-FDG uptake was positively associated with GLUT2, GLUT3, and SGLT1 expression (**Figure 4C**); (5) in mWAT, post-op AUC negatively associated with mWAT GLUT1, GLUT2, and SLGT2 expression, whereas post-op fasting glucose level and weight were negatively associated with mWAT FDG uptake (**Figure 4D**); (6) in rpWAT, RYGB surgery correlated with rpWAT FDG radioactivity, rpWAT GLUT1 expression, and GLUT5 expression (**Figure 4E**), whereas post-op AUC, fasting glucose level, and weight were negatively associated with iWAT FDG uptake and GLUT1 expression. These data are consistent with systemic glucose metabolism changes in RYGB-operated patients and rats. These results indicated that systemic glucose metabolism changes are robustly associated with upregulation of GLUT1, and related transporter genes underwent RYGB.

## Discussion

The biological mechanism of improved hyperglycemia and weight loss by bariatric remains unelucidated. Previous studies focused on the anatomical changes of the intestine or gut oriented hormones, such as glucagon-like peptide 1. Here, we demonstrate that the potential image-based semi-quantitative FDG activity of systemic glycolytic activity correlated with improved glycemic control and robust weight loss brought by RYGB in a rodent model. We analyzed the correlation between increased systemic glucose metabolism and RYGB-induced improvement in metabolic changes. We have shown that (1) the increased glucose metabolism not only in AL and CL of the intestine but also in mWAT, rpWAT, and iWAT; (2) the increased expression of glucose transporter subtypes in organs showed improved glucose metabolism after RYGB; and (3) the correlation between increased organ-specific glucose metabolism and improved hyperglycemia in rats after RYGB.

Insulin-induced glucose disposal occurs in muscle, fat, and various organs in normal physiology (26). After bariatric surgery in obese patients with T2D, micro-PET analysis revealed a possible increase in systemic glucose uptake (**Figures 1**, **2**). FDG uptake was not changed in the muscle, a representative glucose disposed organ, but newly increased in the intestine and various fat tissues. According to the TAC graph, FDG was trapped for over 60 min in both the intestine and fat, unlike in muscles and liver (**Figure 2**). Some studies showed that bariatric surgery leads to a significant improvement in hepatic insulin sensitivity, which is accompanied by increased hepatic glucose uptake and reduced endogenous glucose production in human T2D or non-T2D patients (27). However, our results showed that hepatic glucose uptake was comparable. Several studies demonstrated that FDG uptake in the liver was lower or comparable in RYGB-operated groups (20, 21), which may be caused by a glucose analogue in which FDG cannot be metabolized. It is postulated that Glucose-6-phophatase (G6pase) is expressed in liver and this enzyme may affect the retention of FDG in liver. Also, in the muscle, an earlier FDG uptake surge was observed but preceded a slow washout of FDG radioactivity over the remaining 60 min and showed that insulin-induced glucose disposal does not change. Previous studies showed that bariatric surgery induced improvement in glycemic control independently with insulin secretion or sensitivity (20, 21, 28, 29). Unlike in liver or muscle, FDG retention is maintained in various fat tissues, including mWAT, iWAT, and rpWAT, which are the organ where glucose is uptaken by insulin-induced GLUT4 activity (30). However, our data showed that although GLUT4 expression is comparable, the expression of GLUT1 was increased. This result indicated that the high expression of GLUT1, whose activity is independent of insulin, contributes to increased glucose uptake after RYGB surgery. Similar to reprogrammed intestinal glucose metabolism after RYGB is mediated by GLUT1, GLUT1 contributes to glucose absorption in various fat tissues. Accordingly, systemic reprogrammed glucose absorption patterns were mediated independently of insulin-mediated effects.

Our results indicate that compared with sham-operated rats, GLUT1 is upregulated in organs with increased FDG uptake of RYGB-operated animals. GLUT1 and glycolysis genes are increased in the intestine after bariatric surgery (20, 21, 23, 28, 31). Consistent with previous studies (23, 32), intestinal glucose transporter GLUT2 and SGLT1 expression were downregulated in the AL and CL of RYGB-operated rats. In organs with a high FDG uptake, GLUT1 expression was increased. mWAT, iWAT, and rpWAT showed the increased expression of GLUT1 after RYGB surgery. Conversely, GLUT1 expression was comparable in the organ with no change in FDG uptake after RYGB. It can be inferred that the insulin-independent higher FDG uptake in fat is due to the increase in GLUT1. GLUT1 is rarely expressed in mature intestine and white adipose tissue (33). Intestinal hypertrophy and increasing villus length are caused by intestinal glucose uptake through GLUT1 after RYGB (20, 21, 34, 35). In our previous study, Amphiregulin (AREG)/Epidermal growth factor receptor (EGFR) signaling is upregulated in RYGB rats and human intestine, and AREG increases glucose uptake and secretion through EGFR-mediated GLUT1 expression and trafficking (21). Also, oxygen is insufficient due to numerous build-up processes occurring in the small intestine, which leads to the overexpression of GLUT1 by HIF1-a (36). We speculate that soluble factors such as AREG not only affect the intestine, but also affect the various organs through circulating through the blood. AREG may regulate GLUT1 expression in various fats throughout the body. In fact, it was reported that overexpression of AREG in white adipose tissue of db/db mice reduced mouse weight and fat weight and beta-oxidation signaling and TCA cycle would be increased in white adipose tissue (37). Furthermore, the T Helper cell modulates intestinal stem cell renewal and intestinal morphology (38). Previous studies showed that AREG, which originated from T helper 2 cells, results in the upregulation of the GLUT1 expression (21). Therefore, the intestinal derived signal may affect the glucose disposal of fat tissues independent of insulin action or intestine-derived T helper 2 cells systemically similar to other immune cells (39).

The current study has several limitations. First, we only used ^18^F-fluoro-2-deoxyglucose (FDG). However, glucose is trapped in the cell in the form of glucose-6-phosphate (G6P). G6P is a late limiting step in glucose metabolism. FDG in the intestine and fat tissues was retained for over 60 min; therefore, if glucose had been used instead of FDG, it would have been metabolized. Also, previous study showed that ^14^C-Glucose disposal and excretion was similar to those of FDG. Second, we only showed correlation and not causation through experiments. However, to our knowledge, this study is the first to demonstrate the quantitative measurements of post-RYGB FDG uptake in various organs and show dynamic glucose retention. This study supports the RYGB-induced new systemic glucose distribution. Further studies are needed to identify the signaling pathways inducing GLUT1-mediated systemic glucose disposal, which may be new drug targets to mimic the RYGB-induced improvement in metabolic changes.

In conclusion, the induction of GLUT1 levels by RYGB is robust and is correlated with improved metabolic changes. This novel finding may support the unknown mechanism effect of RYGB.

## Data Availability Statement

The raw data supporting the conclusions of this article will be made available by the authors without undue reservation.

## Ethics Statement

The studies involving human participants were reviewed and approved by Approved by Severance Hospital’s institutional review board. The ethics committee waived the requirement of written informed consent for participation. The animal study was reviewed and approved by Institutional Animal Care and Use Committee (IACUC) at the Yonsei University Health System. Written informed consent was not obtained from the individual(s) for the publication of any potentially identifiable images or data included in this article.

## Author Contributions

Conceptualization: CKu and AC. Data curation and formal analysis: CKu, JO, KP, and AC. Funding acquisition: CKu. Investigation and methodology: CKu, JO, CKa, EW, JN, SL, and AC. Supervision and validation: CKu, JO and AC. Visualization: CKu, JO, and AC. Writing original draft: CKu, JO, and AC. Writing review and editing: CKu, JO, EL, KP, and AC. All authors contributed to the article and approved the submitted version.

## Funding

This research was supported by a grant from the Korea Health Technology R&D Project through the Korea Health Industry Development Institute (KHIDI), funded by the Ministry of Health & Welfare, the Republic of Korea (grant number: HR18C0012).

## Conflict of Interest

The authors declare that the research was conducted in the absence of any commercial or financial relationships that could be construed as a potential conflict of interest.

## Publisher’s Note

All claims expressed in this article are solely those of the authors and do not necessarily represent those of their affiliated organizations, or those of the publisher, the editors and the reviewers. Any product that may be evaluated in this article, or claim that may be made by its manufacturer, is not guaranteed or endorsed by the publisher.
