# Supplementary material for: Altered Glucose Metabolism and Glucose Transporters in Systemic Organs After Bariatric Surgery
Source: Front Endocrinol (Lausanne). 2022 Jul 14;13:937394. doi: 10.3389/fendo.2022.937394 (PMC9329688; doi:10.3389/fendo.2022.937394)
Supplement: Supplementary file 1 [file Table_1.docx]

Supplementary Material

**Table S1.**

| Gene | NCBI accession no. | Sequence |
| --- | --- | --- |
| *GLUT1* | NM_138827 | CTCATAGCTGGACGGCTGTT  GGCCCAAGGTAGCTGGAAAT |
| *GLUT2* | NM_012879 | TCAGCCAAGGACCCCGTCCC  AAGGCCCGAGGAAGTCCGCA |
| *GLUT3* | NM_017102 | GCGCAGCCCTTCCGTTTTGC  CGCTGGAGGATCTCCGTCGC |
| *GLUT4* | NM_012751 | CCGACGGACACCTTCTCTCT  ACCATTTTGCCCCTCAGTCAT |
| *GLUT5* | NM_031741 | TCGCACTGGCACTGCAGAACA  GCCCCACGGCGTGTCCTATG |
| *SGLT1* | NM_013033 | GGGGACTGATTCTCGGCTTC  AGATAGTGGACCCCGCAGAT |
| *SGLT2* | NM_022590 | ATGGAGGGACACGTAGAGGAA  ACCACAAGCCAACACCAATG |
| *HK* | NM_012735 | GAGTACATGGGCATGAAGGG  ACTTCACAGTGAGGGTCTTC |
| *G6pase* | NM_013098 | CCCAGACTAGAGATCCTGACAGAAT  GCACAACGCTCTTTTCTTTTACC |
| *PCK* | NM_198780 | GCCTGTGGGAAAACCAACCT  CACCCACACATTCAACTTTCCA |
| *18S* | NR_046237 | GGAGAGGGAGCCTGAGAAAC  CAATTACAGGGCCTCGAAAG |
